# Successful Simultaneous Peritoneal Dialysis Catheter Removal and Reinsertion During Surgical Repair of Acute Small Bowel Perforation: A Case Report

**DOI:** 10.1155/crin/8739668

**Published:** 2026-04-11

**Authors:** Pannawat Mongkolrattanakul, Chote Jitopas, Vatcharee Chareonphaibul, Kittiphan Chienwichai

**Affiliations:** ^1^ Division of Nephrology, Department of Internal Medicine, Phanatnikhom Hospital, Phanat Nikhom, Chonburi, Thailand; ^2^ Division of General Surgery, Department of Surgery, Phanatnikhom Hospital, Phanat Nikhom, Chonburi, Thailand; ^3^ Clinical Research and Development, Medical Affairs, Vantive Healthcare, Bangkok, Thailand; ^4^ Division of Nephrology, Department of Internal Medicine, Hatyai Hospital, Songkhla, Thailand

**Keywords:** bowel perforation, case report, peritoneal dialysis, simultaneous catheter reinsertion, surgical repair, Tenckhoff catheter

## Abstract

Bowel perforation is a rare but serious complication of Tenckhoff catheter placement for peritoneal dialysis (PD), particularly when blind insertion techniques such as the Seldinger method are used. Standard management typically involves catheter removal, surgical repair of the bowel, and delayed reinsertion of a new catheter following completion of systemic antibiotic therapy. Although this approach minimizes the risk of infection, it necessitates a second surgical procedure and can significantly delay the initiation of PD. We report the case of a 60‐year‐old woman with end‐stage kidney disease secondary to diabetic nephropathy who developed small bowel perforation during Tenckhoff catheter insertion via the Seldinger technique. To avoid a second operation, and in accordance with the patient’s preference, simultaneous catheter removal and contralateral reinsertion were performed during surgical repair of the perforation. The peritoneal cavity was irrigated with 2.5 L of normal saline, a drain was placed, and the patient received a 14‐day course of intravenous meropenem with peritoneal rest. The drain was removed on postoperative Day 5, and automated PD was successfully resumed two weeks later without any evidence of peritonitis. This case suggests that simultaneous catheter removal and reinsertion during bowel repair may be technically feasible under specific, favorable intraoperative conditions, potentially avoiding an additional procedure and facilitating earlier return to PD. However, this approach is not supported by current guidelines, which recommend delayed catheter reinsertion to allow adequate peritoneal healing, and should not be considered standard practice.

## 1. Introduction

Peritoneal dialysis (PD) is a widely used modality of kidney replacement therapy [1], and the Tenckhoff catheter is essential for its delivery. Although catheter insertion is generally safe, bowel perforation, which occurs in less than 1% of cases [2–4], remains a serious complication. Immediate perforations are typically the result of direct mechanical trauma, particularly when blind insertion techniques such as the Seldinger method are employed [5].

Standard management of bowel perforation occurring during Tenckhoff catheter insertion involves removing the catheter responsible for the injury, performing surgical repair of the bowel, and delaying reinsertion of a new catheter for several weeks to months to allow adequate peritoneal healing. This approach often necessitates temporary hemodialysis and an additional surgical procedure, both of which can impose psychological and logistical burdens on the patient. Furthermore, interruptions in PD therapy during this period may increase the risk of early technique failure [6, 7] and transition to long‐term hemodialysis [8].

Here, we describe a case demonstrating the feasibility of simultaneous catheter removal and contralateral reinsertion during surgical repair of acute bowel perforation, followed by successful resumption of PD two weeks postoperatively. This approach eliminates the need for a secondary procedure, reduces the interval to dialysis initiation, and may help preserve PD as the patient’s long‐term dialysis modality.

## 2. Case Presentation

A 60‐year‐old Thai woman with end‐stage kidney disease secondary to diabetic nephropathy was scheduled for Tenckhoff catheter placement in November 2024 following a shared decision‐making process. She had a body mass index of 20 kg/m^2^. The procedure was performed using the Seldinger technique. During intraoperative testing with PD solution, fecal material was unexpectedly observed in the Y‐connector during outflow, immediately raising suspicion of bowel perforation. Despite this finding, the patient remained hemodynamically stable and exhibited no signs of peritonitis.

An urgent surgical consultation was obtained. The patient’s family expressed a strong preference to avoid a second operation and requested that, if feasible, a new catheter be inserted during the same procedure as bowel repair. The potential risks, including peritonitis and catheter dysfunction, were discussed in detail, and informed consent was obtained.

**FIGURE 1:**
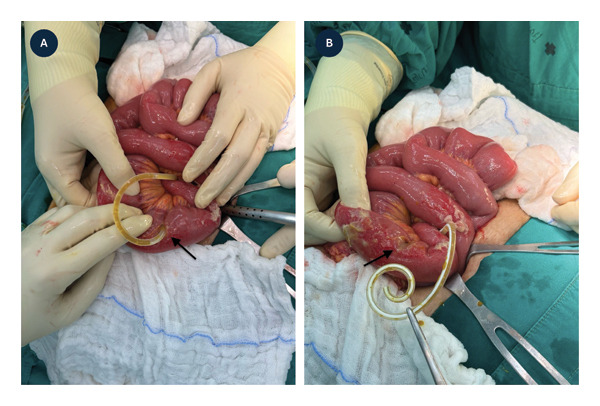
Intraoperative findings of bowel perforation during Tenckhoff catheter placement. (A) Tenckhoff catheter (black arrow) penetrating the small bowel approximately 40 cm proximal to the ileocecal valve. (B) Perforation site (black arrow) after catheter removal, prior to primary surgical repair.

Exploratory laparotomy revealed that the catheter had penetrated the small bowel approximately 40 cm from the ileocecal valve, without evidence of gross peritoneal contamination, as shown in Figure 1. Primary repair of the perforation was performed using a two‐layer transverse closure. The peritoneal cavity was extensively irrigated with 2.5 L of normal saline, and a surgical drain was placed. After removal of the contaminated catheter, a new Tenckhoff catheter was inserted on the contralateral side.

Postoperatively, the patient received intravenous meropenem for 14 days. Meropenem was selected because the patient had been hospitalized two months earlier for bacterial pneumonia and had received ceftriaxone, raising concern for colonization with extended‐spectrum beta‐lactamase (ESBL)–producing organisms. Oral fluconazole 200 mg every 48 h was administered throughout the antibiotic course as antifungal prophylaxis, in accordance with ISPD recommendations [9]. Peritoneal rest was maintained for two weeks. The surgical drain was removed on postoperative Day 5 without evidence of infection.

Throughout the postoperative period, she remained afebrile and hemodynamically stable, without abdominal pain or tenderness, and serial laboratory evaluations showed no leukocytosis. During the two‐week peritoneal rest period, temporary hemodialysis was not required. The patient maintained adequate urine output, and metabolic parameters, including serum potassium, acid–base balance, and volume status, remained stable with conservative medical management.

Automated PD was resumed two weeks after surgery. At that time, the dialysate effluent was clear, with a normal leukocyte count and differential and a negative bacterial culture. Repeat effluent analysis performed two weeks after resumption of PD remained within normal limits, with no neutrophilic predominance. The patient remained free of peritonitis during 10 months of follow‐up.

**FIGURE 2:**
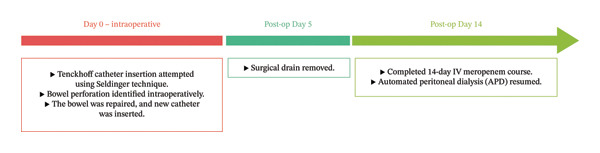
Timeline of clinical events from Tenckhoff catheter insertion through postoperative management. The figure illustrates the sequence of key clinical events: initial catheter insertion via Seldinger technique, intraoperative detection of bowel perforation, simultaneous surgical repair with catheter removal and contralateral reinsertion, postoperative antibiotic therapy with peritoneal rest, drain removal on Day 5, and successful resumption of automated peritoneal dialysis at 2 weeks.

The catheter demonstrated satisfactory inflow and outflow functions without dialysate leakage or other mechanical complications. No catheter manipulation or revision was required. Dialysis delivery was adequate, with stable volume status and satisfactory metabolic control. The clinical course is summarized in Figure 2, which illustrates the timeline of key events from Tenckhoff catheter insertion through surgical repair and subsequent postoperative management.

## 3. Discussion

Bowel perforation is an uncommon but potentially life‐threatening complication of PD catheter insertion, with an incidence of less than 1% [2–4]. The risk is particularly heightened when blind techniques such as the Seldinger method are used, as direct mechanical trauma to the bowel can occur without immediate clinical signs [5]. Early recognition is therefore essential, as delayed diagnosis can lead to peritonitis, sepsis, and adverse outcomes.

Standard management following bowel perforation typically involves removal of the catheter, surgical repair of the perforation, and a prolonged course of systemic antibiotics, with delayed reinsertion once the peritoneum has healed [8, 10, 11]. This approach minimizes the risk of infection but often requires temporary hemodialysis and an additional procedure for catheter reinsertion. Such delays can disrupt the patient’s PD program, contribute to early technique failure [6, 7], and increase the likelihood of permanent transition to hemodialysis [8]. Furthermore, the need for a second procedure carries additional surgical risks and logistical burdens for both patients and healthcare systems.

In contrast, simultaneous catheter removal and reinsertion is an established strategy in other PD‐related settings, including unresolved exit‐site or tunnel infections [12] and antibiotic‐responsive relapsing, recurrent, or repeat peritonitis [9]. However, its role in the setting of acute visceral perforation has not been well described. To evaluate whether this approach has been previously reported, we conducted a focused literature search in PubMed and Google Scholar from database inception to December 2025 using combinations of relevant terms related to PD, Tenckhoff catheter, bowel perforation, and catheter reinsertion. We did not identify any published reports describing catheter replacement performed during surgical repair of acute bowel perforation. This case adds to the limited literature by demonstrating that simultaneous catheter replacement during bowel repair can be both safe and effective when performed under carefully selected circumstances.

In our institution, percutaneous catheter insertion using the Seldinger technique is commonly performed in patients without prior abdominal surgery because it is minimally invasive and resource efficient. The patient had no history of prior abdominal surgery or clinical suspicion of intra‐abdominal adhesions, so preoperative imaging was not performed. Although laparoscopic or open placement allows direct visualization and may reduce the risk of visceral injury, particularly in patients with prior surgery or suspected adhesions, the Seldinger technique remains widely practiced in appropriately selected patients [13–15].

Several key factors likely contributed to the favorable outcome in our patient. First, bowel injury was promptly recognized intraoperatively, minimizing the degree of peritoneal contamination. Second, meticulous surgical technique, including primary two‐layer closure of the perforation and extensive peritoneal lavage, helped reduce the risk of postoperative infection. Third, contralateral placement of the new Tenckhoff catheter avoided previously contaminated tissue planes and reduced the likelihood of mechanical complications. Finally, the combination of systemic antibiotics and peritoneal rest allowed adequate peritoneal healing before dialysis resumption.

This case illustrates the value of shared decision‐making in PD management. The patient’s preference to avoid a second surgery was incorporated into clinical planning, an approach that may enhance satisfaction [16], adherence [17], and long‐term PD continuation [18]. However, the clinical decision to proceed with simultaneous reinsertion was ultimately based on the surgical team’s intraoperative assessment that the specific conditions—absence of gross peritoneal contamination, successful primary bowel repair, and hemodynamic stability—provided a reasonable basis for attempting this approach. We acknowledge that shared decision‐making involves supporting patients in making informed decisions based on a clear understanding of risks and benefits and does not oblige clinicians to perform procedures that carry a disproportionately high risk of failure or that could compromise patient safety.

This case demonstrates that simultaneous catheter reinsertion during bowel perforation repair is technically feasible, provided that specific favorable conditions are fulfilled, including immediate recognition of bowel perforation, absence of gross peritoneal contamination, hemodynamic stability, and successful primary repair of the perforation. However, it is essential to highlight that current guidelines do not favor this approach [9, 12]. Current recommendations advocate a staged approach to allow adequate peritoneal healing, clear potential infection, and assess for adhesion formation before placing a new catheter. Moreover, a delayed laparoscopic approach for catheter reinsertion would have been the most guideline‐concordant strategy, as it allows direct visualization of the peritoneal cavity to exclude adhesions and ensure a safe insertion site, particularly important in a patient who has undergone laparotomy for bowel repair. As this is a single case, the findings cannot be generalized, and the favorable outcome should not be interpreted as justification for routine practice. We acknowledge that deliberate evaluation of this approach through prospective interventional studies would not be ethically justified. Future case reports or retrospective case series documenting similar clinical scenarios may help further characterize patient selection criteria and long‐term outcomes.

## 4. Conclusion

Simultaneous catheter removal and reinsertion during surgical repair of bowel perforation is technically feasible under specific favorable intraoperative conditions, as demonstrated in this case. This approach may avoid additional procedures and facilitate earlier return to PD in carefully selected patients. However, current guidelines recommend delayed catheter reinsertion to allow adequate peritoneal healing and infection clearance, and this approach should not be considered standard practice. Future case reports or retrospective case series may help further define patient selection criteria and long‐term outcomes.

## Funding

No funding was obtained for this study.

## Disclosure

Vantive Healthcare had no role in the patient’s clinical management, study design, data analysis, manuscript preparation, or the decision to submit the manuscript for publication.

## Consent

Written informed consent was obtained from the patient for the publication of this study.

## Conflicts of Interest

Vatcharee Chareonphaibul is an employee of Vantive Healthcare (Thailand) Ltd. She contributed to data curation and manuscript review as an individual author. The other authors declare no conflicts of interest.

## Patient Perspective

“I was worried when the doctors told me about the bowel injury and the possibility of another surgery later. I am grateful that they were able to repair it and place a new catheter in the same operation, allowing me to start dialysis as planned without undergoing additional procedures.”

## Data Availability

All data relevant to the case are included within the article.
